# Myomectomy during Caesarean Birth in Fibroid-Endemic, Low-Resource Settings

**DOI:** 10.1155/2013/520834

**Published:** 2013-11-14

**Authors:** J. O. Awoleke

**Affiliations:** Department of Obstetrics and Gynaecology, Ekiti State University Teaching Hospital, Ado-Ekiti, Ekiti State P.M.B. 5355, Nigeria

## Abstract

If myomectomy during caesarean delivery becomes a widespread practice, it could potentially eliminate multiple surgeries for both indications. However, many surgeons have been reluctant to adopt this policy without conclusive evidence demonstrating its safety. This study reviews the publications on caesarean myomectomy especially from the African Continent with respect to duration of surgery, blood loss, length of hospital stay, and blood transfusions. Judging from the lack of large studies on caesarean myomectomy, the proportion of surgeons who attempt the procedure is largely low because of concerns about its safety. However, most of the authors suggested that the complications and morbidity following caesarean myomectomy do not significantly differ from those occurring during caesarean section alone, while fertility is apparently not compromised by this treatment. With careful patient selection, adequate experience, and efficient haemostatic measures, the procedure does not appear as hazardous as was once thought. This piece of information is relevant for counseling women who request for the simultaneous removal of previously diagnosed fibroids during caesarean section. Staff and facilities for safe management of haemorrhage are a requisite for the procedure. Large randomized trials are needed to guide decisions as to the best clinical practice regarding myomectomy during caesarean delivery.

## 1. Introduction

Fibroids are benign tumours of the uterine smooth muscle commonly found in a great proportion of women who live on the African Continent [1]. Although the exact incidence is unknown, they generally affect women in the childbearing ages. At postmortem, an incidence of about 50% of women has been documented [2], while they have been encountered in 0.3% to 3% of pregnancies [3, 4].

These tumours may be asymptomatic. In the pregnant women with coexisting fibroids, there are increased incidences of first trimester losses, pressure symptoms, pain from red degeneration (necrobiosis), torsion of a pedunculated variant, malpresentations, preterm rupture of membranes and preterm labour [3, 5] during *pregnancy*, obstructed labour from a cervical or lower segment mass *intrapartum* and retained placenta, subinvolution of the uterus, postpartum endomyometritis, and postpartum haemorrhage in the immediate *postpartum* period [3, 5–7]. 

Management includes medical and surgical options, although the definitive and commonest treatment modality is still hysterectomy [8]. However, in the African setting where many women have an aversion to sterilizing surgeries and high premium is placed on children and childbearing, fertility-preserving options will still be favoured [9, 10]. 

Thus, there exists the possibility of the same woman undergoing a myomectomy and, later, a caesarean section or *vice versa*. If these two procedures can be safely performed at the same time, the risk of anaesthetic complications, multiple surgeries, adhesions and intra- or postoperative haemorrhage, exorbitant costs of operative procedures, and hospital stay could be reduced. This may provide a leeway in the management of this gynaecological disorder in the developing nations of Africa, the very population in which it is the commonest.

This study was thus conducted to examine the evidence, if any, for the safety of myomectomy during caesarean section.

## 2. Materials and Methods

An electronic search of the published literature was conducted using the search terms “caesarean myomectomy,” “myomectomy during caesarean section,” and “fibroids and pregnancy.” PubMed and other indexed journals in the field of obstetrics and gynaecology that specifically addressed fibroids coexisting with pregnancy were searched. Websites of international organizations and private foundations with information on the management of pregnant women with fibroids were also searched. Original and review articles along with case reports that addressed the subject matter were included in the study.

## 3. Results

### 3.1. History

Elective myomectomy at the time of caesarean section has been traditionally discouraged due to the attendant morbidity, primarily from haemorrhage. Although there appears to be little direct evidence, the removal of intramural myomata from the pregnant uterus was regarded as unsafe because of the recognized difficulty in controlling blood loss [11]. 

However, two of the earliest works on caesarean myomectomy included a report of 13 surgeries with successful outcomes (although one patient had intraoperative haemorrhage requiring uterine artery ligation and blood transfusion) [12] and a review of 47 incidental myomectomy cases during caesarean section which noted that, although the procedure added 11 minutes to the time for a caesarean section, 112 mLs to the blood loss at surgery, and half-a-day to the duration of hospital stay, there were no wound infections or serious morbidity [13]. Both suggested that concomitant myomectomy with caesarean section was safe if it was done in carefully selected patients.

Subsequent documentation showed that, out of a small series of 9 patients who underwent elective myomectomy at the time of caesarean delivery, 3 were reportedly complicated by severe haemorrhage requiring obstetric hysterectomy [14], while, in another report, out of 5 cases, 4 were performed on pedunculated fibroids and were removed without difficulty, while the only nonpedunculated fibroid that was removed was associated with severe hemorrhage [15].

In the last decade, there have been various reports using several indices to assess the safety or otherwise of this procedure. 

### 3.2. Indications

Documented reasons for the removal of uterine fibroids during caesarean section include the prevention of necrobiosis [16], pain during pregnancy, and unusual intraoperative appearance of the tumour [17], to gain access to the baby in patients in whom fibroids are obstructing the lower uterine segment [18], with pedunculated and anterior uterine fibroids [17, 19, 20], and when the fibroids cause difficulty with uterine wound closure thereby causing significant blood loss [6, 21].

### 3.3. Type of Uterine Incisions

One study included caesarean sections done using the lower segment transverse incision in 105 out of 111 (94.6%) cases and 250 out of 257 (97.3%) controls (who had caesarean section without myomectomy), while the classical incision was employed in the remainder of the subjects [17]. An Israeli study noted that, in four out of 32 cases (12.5%), the caesarean sections were classical, and the reminder were done via a lower segment incision [22].

However, the myomectomy incisions were generally made on the anterior uterine wall [1, 19].

### 3.4. Sites and Sizes of the Fibroids

See Table 1.

### 3.5. Haemostatic Measures

Several recent studies have described techniques which can minimize blood loss at caesarean myomectomy. These include the application of a tourniquet to encompass and compress both uterine arteries at the base of the broad ligament and the vessels in the infundibulopelvic ligament after lifting away the Fallopian tubes, thus creating a relatively bloodless operating field [1, 23], bilateral uterine artery ligation [23], electrocautery [24], high-dose oxytocin infusion during (i.e., after the delivery of the baby and placenta) and after the surgery [22, 25, 26], and a combination of uterine tourniquet and high-dose oxytocin infusion [21].

Sapmaz et al. [23] randomized 52 women with fibroids coexisting with pregnancy that had caesarean myomectomy into two groups: the first had bilateral ascending uterine artery ligation, while the second had intraoperative tourniquet applied to assist with haemostasis. Total intraoperative blood loss, total operation duration, number of enucleated myomata, and febrile morbidity were similar in the 2 groups. However, urgent laparotomy and bilateral internal iliac artery ligation had to be performed in 1 patient in the tourniquet group because of postoperative hemorrhage. They concluded that, despite the fact that bilateral ascending uterine artery ligation and tourniquet use had similar outcomes with regard to intraoperative blood loss in caesarean myomectomy cases, the efficacy of ligation on blood loss in the postoperative period continues owing to its permanence.

### 3.6. Perioperative Issues

See Table 2.

When the size of the uterine myomata exceeded 6 cm, the operation time was observed to be longer in the caesarean myomectomy group [27]. Other documented perioperative concerns during caesarean myomectomy include postoperative anaemia, fractures of the humerus and clavicle in one baby, and puerperal sepsis [19, 28]. Repeat operations following caesarean myomectomy have been documented mostly for excessive bleeding and one for haematoma formation below the scar [22]. These have sometimes resulted in hysterectomy. A retrospective case-controlled study including 1,242 pregnant women with fibromyomas who underwent myomectomy during caesarean section and three control groups of 200 matched pregnant women without fibromyomas who underwent caesarean deliveries (Group A), 145 patients with fibromyomas who underwent caesarean deliveries without removal of fibromyomas (Group B), and 51 patients with fibromyomas who had a hysterectomy during caesarean section found no differences in the mean hemoglobin change, the incidence of postoperative fever, and the length of hospital stay among all of the groups [29]. 

### 3.7. Future Obstetric Performance

In a follow-up study of 25 women who had caesarean myomectomy in their erstwhile pregnancies, three (12%) patients became pregnant, two (66.7%) of whom had normal vaginal deliveries, while the third had a repeat elective caesarean section [28]. A prospective nonrandomized study was conducted by Adesiyun et al. [30] to determine the future fertility and pregnancy outcomes in 29 pregnant clients who had caesarean myomectomy in their last delivery. Of these, 22 (75.9%) of the females have had only one parous experience, and 25 (86.2%) had had one previous caesarean section. Only six (20.7%) of them had fertility treatment before the attainment of the index pregnancy. The common antenatal complications recorded were abnormal lie/malpresentation (10.3%), placenta praevia (10.3%), and threatened abortion (10.3%). They found 17 patients eligible for trial of vaginal birth out of which 13 (44.8%) had successful vaginal births after caesarean myomectomy. Of the 16 (55.2%) that had repeat caesarean section, 1 also had caesarean hysterectomy. There was no maternal or perinatal mortality recorded. They concluded that future fertility and/or subsequent pregnancy outcome in patients is unaffected by caesarean myomectomy. 

## 4. Discussion

Fibroids are the commonest tumours in our female population in Nigeria. In fact, Agboola [31] reckoned that over 80% of women above 25 years have fibroids, even if they were just the size of a seedling! Hardly is the operation list of a gynaecologist in the country complete without the inclusion of myomectomy or hysterectomy for the removal of fibroids. Also, fibroid(s) coexisting with and/or complicating pregnancy is not an uncommon presentation to obstetricians practicing in Africa [19].

Uterine fibroids could become symptomatic, and, then, therapy becomes inevitable. However, in most countries on the African Continent where fibroids are common, the most popular management options are “conventional” myomectomy and hysterectomy. This is because the other alternatives available in the developed nations appear to be more expensive, with infrastructural support way beyond the capability of many African nations. The training/expertise is also not widely available in most parts of the continent.

Many African women, however, have an aversion for surgeries resulting in loss of the womb because of the cultural and, at times, religious belief of reincarnation (without their wombs!) and an attachment to preservation of menstruation and childbearing [9, 10].

Also, caesarean section has become one of the commonest hospital-based operative procedures in many African nations. For example, in Ghana, the rate increased from 10% in 1970 to 20% in 1999 [1]. 

If myomectomy could be safely done during caesarean deliveries, it could prevent the added morbidity of a separate procedure (laparotomy to remove fibroids, anaesthesia, and its possible complications) in the future, justifying the cost effectiveness of the approach [25]. This would be a significant benefit of the procedure in resource-constrained settings [3]. Puerperal uterine subinvolution could also be minimized as well as other known complications of fibroids such as menorrhagia, anaemia, and pain (e.g., from torsion or “red” degeneration during a subsequent pregnancy) [1]. Also, since fibroids are sometimes located in the lower uterine segment, their nonremoval will only leave the surgeon with one alternative: a “classical” incision on the uterus with all of its attendant complications. 

Apart from the above-stated advantages of caesarean myomectomy, another benefit is that it increases the chances of vaginal delivery in subsequent pregnancies when removed from the lower uterine segment [32]. The scar integrity following caesarean myomectomy has been shown to be better than that following interval myomectomy [33, 34] when assessed with serial ultrasound scan in subsequent pregnancies [33] and at subsequent caesarean section [34].

The results from the reviewed studies indicate that caesarean myomectomy is safe and offers no significantly increased risks to the patient than caesarean section alone. These surgeries have been largely performed on pedunculated and anterior fibroids individually less than 6 cm and those obstructing the lower uterine segment or wound closure after extraction of the baby. However, if it is performed as an emergency procedure when the patient is already in labour or has ruptured the fetal membranes, there is an increased risk of sepsis occurring [19]. 

The problem of haemorrhage with the need for blood transfusion, especially in our environment where unsafe blood transfusion due to transmission of Human Immunodeficiency Virus (HIV) and serum Hepatitides still occurs, is also of paramount concern [35]. Improving the outcome of this procedure would also require improved blood-banking services.

## 5. Conclusion 

The possibility of safely performing myomectomy during caesarean section is appealing in the low-resource settings of the sub-Saharan Africa where fibroids are common. 

The decision to proceed with elective myomectomy at the time of caesarean delivery should be approached with caution and should perhaps be limited only to patients with pedunculated fibroids or to instances in which the lower segment incision (for extracting the baby) cannot be closed without removal of the fibroid(s). Centres in which caesarean myomectomy will occur routinely must of necessity have adequately staffed and equipped blood banks whose practices meet international standards.

It is recommended that large multicentre randomized trials be conducted to evaluate the best practice for myomectomy at caesarean section. These will identify appropriate selection criteria, surgical techniques, and haemostatic options and improve the overall outcome of the procedure. Meta-analyses should be done to assess whether other haemostatic options employed on nongravid uteri, for example, vaginal misoprostol, bupivacaine-epinephrine injection, and enucleation of the myomata by morcellation [36], may be effective during caesarean myomectomy. Longitudinal studies to evaluate the long-term obstetric consequences and the risk of uterine rupture in subsequent pregnancies are also needed.

## Figures and Tables

**Table 1:** 

Characteristics	Authors				
	Roman and Tabsh [17]	Kwawukume [1]	Gbadebo et al. [19]	Dimitrov et al. [37]	Ehigiegba et al. [28]
No. of cases (controls)	111 (257)	12 (12)	22	21 (162)	25

Position of fibroids					
Upper segment/corpus uteri		85%	45.5% (10)	63%	
Lower segment		15%	27.3% (6)	23%	
Both segments			27.3% (6)		

Types					
Pedunculated	23%		52.2% (24)		
Subserous	24%				94.8%
Intramural	24%	85%	34.8% (16)	46%	
Submucous	5%		13% (6)		5.2%
Multiple sites	18%				

Sizes					
Mean (range)	Median 3.5 cm (0.9–30 cm)	6 cm			
Value (number/%)	<3 cm (40/36%); ≥3–<6 cm (46/41.4%); ≥6 cm (22/19.8%)		6–10 cm (32/69.9%)		2–10 cm
Nodules removed		1–6 per patient	46	1/patient (85%)	84

**Table 2:** 

Outcome measures	Subjects	Roman and Tabsh [17]	Brown et al. [25]	Hassiakos et al. [38]	Kwawukume [1]	Kaymak [39]
	Cases	111	16	47	12	40
	Controls	257	16	94	12	80

Blood loss	Cases	12.6%	495 mLs (200–1000)			12.5%
	Controls	12.8%	355 mLs (150–900)			11.3%
	*P* value	*0.95 (NS) *	*NS *		*NS *	*NS *

Change in Hb/PCV	Cases	Mean = 5.5%	1.7 g/dl		Mean = 1.83 g/dl	
	Controls	Mean = 6.1%	1.4 g/dl		Mean = 1.73 g/dl	
	*P* value	*NS *	*NS *	*NS *	*NS *	

Blood transfusion	Cases	1/111		NIL	NIL	
	Controls	3/257		NIL	NIL	
	*P* value	*NS *	*NS *			*NS *

Mean operating time (mins)	Cases	55	NA	15 mins longer than control	62.08	NA
	Controls	51	NA		50.83	NA
	*P* value	*NS *	*NA *	*NS *	*NS *	*NA *

Postoperative fever	Cases	4.5%		NA	NA	
	Controls	4.7%		NA	NA	
	*P* value	*NS *	*NS *	*NA *	*NA *	*NS *

Hospital stay (days)	Cases	Mean = 3.6			5 days	
	Controls	Mean = 3.4			5 days	
	*P* value	*NS *	*NS *	*NS *		

mLs: milliliters; NS: not significant (*P* > 0.05); Hb: haemoglobin concentration; PCV: packed cell volume; g/dl: grammes per deciliter; mins: minutes; NA: not assessed.
